# Photon-Counting CT Angiography Enables Superior Preoperative Perforator Depiction for Fibular Transplant Surgery Requiring Less Contrast Agent Compared to Energy-Integrating CT

**DOI:** 10.3390/diagnostics16050798

**Published:** 2026-03-08

**Authors:** Ramin Saam Dazeh, Jan-Lucca Hennes, Tobias Prester, Viktor Hartung, Henner Huflage, Andreas Vollmer, Thorsten Alexander Bley, Philipp Gruschwitz, Kristina Krompaß

**Affiliations:** 1 Department of Diagnostic and Interventional Radiology, University Hospital of Würzburg, 97080 Würzburg, Germany; 2 Clinic and Polyclinic for Oral and Maxillofacial Surgery, Head and Neck Surgery, University Hospital of Würzburg, 97080 Würzburg, Germany

**Keywords:** PCD-CT angiography, image quality, preoperative procedure planning, fibular perforator arteries

## Abstract

**Background/Objectives**: The objective of this study was to ascertain whether photon-counting CT angiography (PCD-CTA) can optimize image quality for the visualization of perforating arteries for planning fibular transplant procedures in comparison to energy-integrating CT angiography (EID-CTA). **Methods**: In this retrospective single-center study, all patients who underwent preoperative CT of the peripheral runoff for planning between October 2021 and July 2023 were consecutively included. PCD-CTA was performed in standard resolution mode as 55 keV images with 90 mL of iodine-containing contrast agent or alternatively, an EID-CTA as a low-kV scan with 110 mL of contrast agent. The raw data were reformatted using comparable soft vascular and sharp regular convolution kernels, slice thickness/increment, and field of view. Contrast-to-noise ratio was calculated for objective image quality. Subjective evaluation was based on a rating by three radiologists using a five-point Likert scale (criteria: overall image quality, luminal attenuation, vessel sharpness, and perforator depiction). **Results**: Of the 26 patients who were screened, 9 could be included in each group, while 8 were excluded due to incomplete reconstructions. The reduction in contrast agent dose resulted in a non-significant decrease in luminal attenuation on PCD-CTA (452.5 ± 53.6 HU vs. 465.5 ± 99.6 HU; *p* = 0.375). The image noise was considerably lower for PCD-CTA (21.1 ± 1.0 HU vs. 32.9 ± 1.6 HU; *p* < 0.001). This resulted in a significantly higher contrast-to-noise ratio (CNR) for sharp kernel reconstructions (22.4 ± 3.5 vs. 14.5 ± 3.8; *p* < 0.001). No significant differences were observed for the soft vascular kernel. Subjective evaluation revealed a significant enhancement in overall image quality, vascular sharpness, and perforator depiction for PCD-CTA with sharp reconstructions. In contrast, soft kernel reconstructions and luminal attenuation demonstrated no substantial difference. Interrater agreement was good to excellent. **Conclusions**: PCD-CTA with sharp kernel reformatting has been demonstrated to yield superior image quality and perforator delineation of the fibular artery in comparison to standard EID-CTA.

## 1. Introduction

Free fibula transplantation (FT) including the adjacent soft tissue is one of the surgical techniques employed in the domain of oral and maxillofacial surgery for the repair of defects that result from surgical procedures on the facial skull. This necessitates meticulous preoperative planning of the graft harvest, predicated on CT angiography of the lower extremity [1,2,3,4,5,6,7]. The visualization of the soft tissue-supplying perforator arteries of the fibular artery (FPA) must be achieved with the utmost precision to facilitate preoperative three-dimensional planning of the incisions and/or the fabrication of three-dimensional-printed templates [8,9,10,11]. Since the commercial market debut of the photon-counting computed tomography (PCD-CT) system, its superiority to conventional energy-integrated computed tomography (EID-CT) regarding radiation dose and contrast agent requirements has been investigated. Its enhanced contrast-to-noise ratio has been demonstrated to significantly improve image quality in comparison with EID-CT, without the necessity of additional ionizing radiation or contrast agents [12,13,14,15]. However, the diagnosis of the peripheral arteries of the lower leg has not yet been sufficiently investigated. Examination and post-processing protocols are primarily based on coronary imaging findings and cannot be applied to peripheral arteries without modification due to the significantly larger scan volume [12,16,17]. Research using a dedicated human body donor model served to elucidate the merits of the aforementioned advantages of the PCD-CT, particularly in the context of imaging peripheral arteries and the influence of various examination parameters on the efficacy of PCD-CT angiography (PCD-CTA) [18,19,20]. Likewise, initial prospective patient studies demonstrated improved diagnostic quality of PCD-CT compared to EID-CT [21,22,23,24,25,26,27].

However, the findings indicate the presence of unexplored possibilities for enhancement, underscoring the necessity for additional research to ascertain the full scope of potential improvements. There is a paucity of examination protocols that fully leverage the capabilities of this advanced detector technology, particularly with respect to the visualization of very-small-caliber vessels, such as the perforator arteries of the fibular artery. This study aimed to determine the current status of image quality for visualizing perforating arteries when planning fibular transplantation using PCD-CTA instead of EID-CT angiography.

## 2. Materials and Methods

### 2.1. Study Design and Ethical Compliance

The ethics committee of the University of Wuerzburg has granted approval for this Declaration of Helsinki-conforming single-center retrospective study (2025-293-dvdh; approval date: 8 January 2025). Patient consent was waived due to the retrospective nature of the study by the Institutional Review Board.

### 2.2. Patient Selection

**Figure 1:**
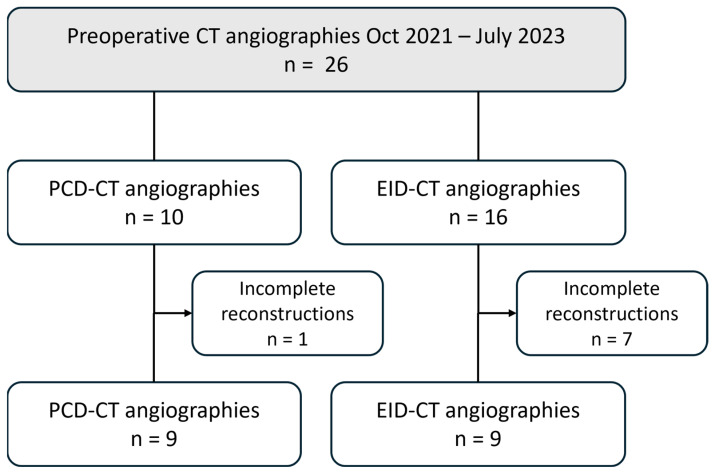
Flowchart illustrating the patient selection process. EID-CT, energy-integrating CT; PCD-CT, photon-counting CT.

All patients who underwent CT angiography of the lower extremities between October 2021 and July 2023 for preoperative planning of a free fibula transplant were retrospectively included in the study. The assignment to PCD-CT or EID-CT was based on scanner capacity on the day of the examination, with no additional criteria applied. The following exclusion criteria were defined: existing or potential pregnancy; underage patients; and incomplete datasets (missing or deviating reconstructions). Figure 1 presents a flowchart that delineates the patient selection process.

### 2.3. CT Angiography Protocols

The PCD-CT examinations were performed using a first-generation photon-counting detector CT (Naeotom Alpha, Siemens, Forchheim, Germany) in spectral standard resolution mode as a 140 kVp scan with an image quality index (IQL) of 156, with a reference tube current of ~50 mAs. The EID-CT examinations were performed using a third-generation energy-integrating detector CT (Somatom Force, Siemens, Germany) as a low-kV scan with automated tube voltage selection of 70/80/90 kVp and corresponding reference tube current of 285/171/120 mAs in single-source single-energy mode. PCD-CT scans extended from the diaphragm to the foot, whereas EID-CT scans only cover the area from the iliac bifurcation to the foot. Therefore, the applied radiation dose is compared using the CTDI_Vol_ of an axial image representative of the femoral head level. Table 1 provides a comprehensive overview of all scan parameters.

The administration of iodinated contrast agent (Imeron^®^ 350 mg iodine/mL, Bracco, Milan, Italy) and 40 mL of post-bolus saline solution via cubital access was performed using a high-pressure injector (CT motion, Ulrich GmbH, Ulm, Germany) at a flow rate of 3.0 mL/s. For the PCD-CT scans, a contrast agent volume of 90 mL (31.5 g iodine) and a volume of 110 mL (38.5 g iodine) were administered for the EID-CTs, with an equal iodine delivery rate of 1.05 gI/s.

### 2.4. Image Reconstruction

The raw data were then reconstructed according to established clinical protocols in a field of view encompassing both legs, with a slice thickness/increment of 1.0 mm using comparable convolution kernels and iterative reconstruction algorithms. Quantum iterative reconstruction (QIR) Level 3 was employed for the PCD-CT, and advanced modeled iterative reconstruction (ADMIRE) Level 3 was utilized for the EID-CT. A soft vascular kernel (Bv36; ρ_50_ 3.38 line pairs/cm) was employed as the convolution kernel for both scanners. Furthermore, reconstructions were performed with a standard body kernel for the PCD-CT scans (Br60; ρ_50_ 8.65 line pairs/cm) and for the EID-CT (Br64; ρ_50_ 10.12 lp/cm) as the best matching alternative. The PCD-CT images were generated spectrally as virtual monoenergetic images with 55 keV, in accordance with the manufacturer’s recommendation. Polyenergetic images were employed for the EID-CT as imperative for low-kV scans.

### 2.5. Quantitative Image Analysis

To perform an objective analysis of image quality, measurements of luminal attenuation (HU_artery_) were taken in the following vessel segments, where possible in both leg arteries: the distal third of the superior femoral artery, the popliteal artery in segment II, the tibiofibular trunk, and all three lower leg arteries in the middle third of the vessel. In the respective image slice, the attenuation in muscle tissue was measured as a measure of background enhancement (HU_muscle_) and the standard deviation of HU values in fat tissue as a surrogate of image noise (SD_fat_) using defined regions of interest (ROIs). The arterial ROIs were set as large as possible, excluding the vessel wall. ROI sizes of 10 mm^2^ were employed for both muscle and fat tissue. Subsequently, the contrast-to-noise ratio (CNR) for each limb was calculated using the following formula: CNR = (HU_artery_ − HU_muscle_)/SD_fat_.

### 2.6. Qualitative Image Analysis

The subjective assessment of image quality was conducted by three radiologists (one senior physician with nine years of experience; two residents with three and one year of experience, respectively). A total of four categories were assessed using a 5-point Likert scale (5 = excellent; 4 = good; 3 = moderate; 2 = fair; 1 = poor): overall image quality; luminal attenuation; vessel sharpness; and depiction of the perforator arteries.

### 2.7. Statistics

Statistical analyses were performed using dedicated software (numiqo Team 2026: Online Statistics Calculator. numiqo e.U., Graz, Austria). The measured values were tested for normal distribution using the Kolmogorov–Smirnov test. Metric data that was normally distributed was reported as mean ± standard deviation, and metric data that was not normally distributed was reported as median and interquartile range (IQR). Significance was tested using the Bonferroni-corrected *t*-test for independent samples or the Friedman test, respectively. Interrater agreement was examined using Kendall’s W. Coefficients below 0.5 were considered insufficient, those between 0.5 and 0.7 indicated good agreement, and those above 0.7 were presumed to indicate excellent agreement [28]. Significance was assumed for *p*-values < 0.05.

## 3. Results

### 3.1. Patient Characteristics, Radiation and Contrast Agent Dosage

Of the 26 patients examined during the study period, 8 were excluded due to incomplete reconstructions (PCD-CT: *n* = 1; EID-CT: *n* = 7). Half of the patients underwent a PCD-CTA examination (mean age: 64 ± 6 years; two females), and the other half underwent an EID-CTA (mean age: 63 ± 5 years; four females).

The mean radiation dose administered during PCD-CTA procedures was found to be largely comparable to that of EID-CT (CTDI_Vol_-PCD-CT: 7.07 ± 0.90 mGy vs. EID-CT: 6.40 ± 2.97 mGy; *p* = 0.491). In all cases, the contrast agent utilized for PCD-CT examinations was 18% less than that employed for EID-CT angiography (90 mL instead of 110 mL).

### 3.2. Quantitative Image Analysis

**Figure 2:**
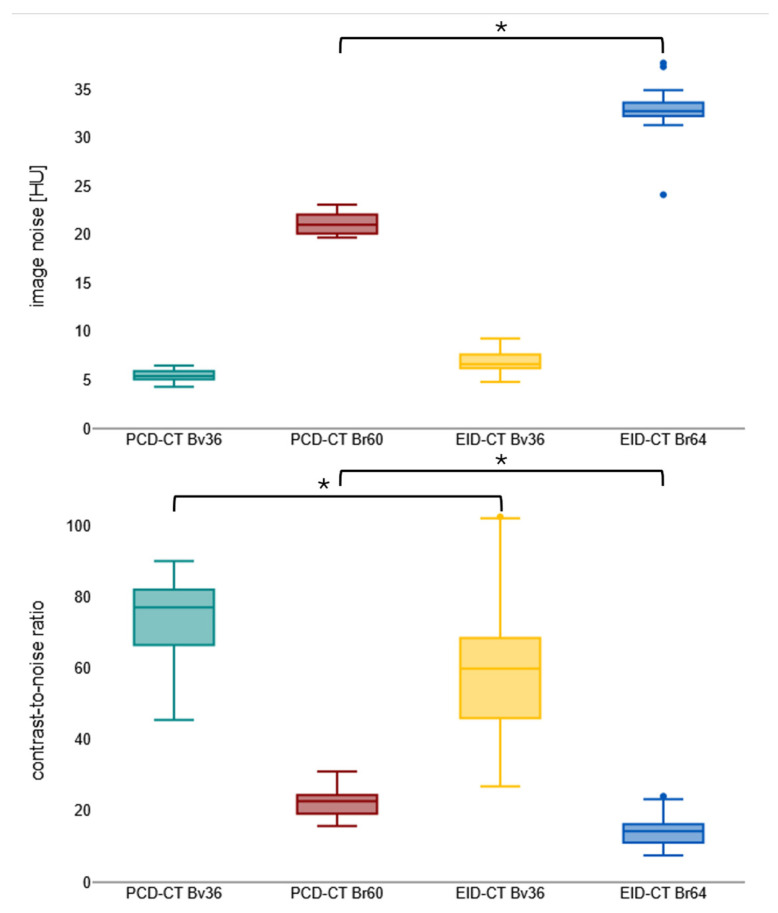
Boxplot visualizing image noise and contrast-to-noise ratio. Bv, body vascular kernel; Br, body regular kernel; EID-CT, energy-integrating CT; HU, Hounsfield units; PCD-CT, photon-counting CT. * *p* < 0.001.

**Figure 3:**
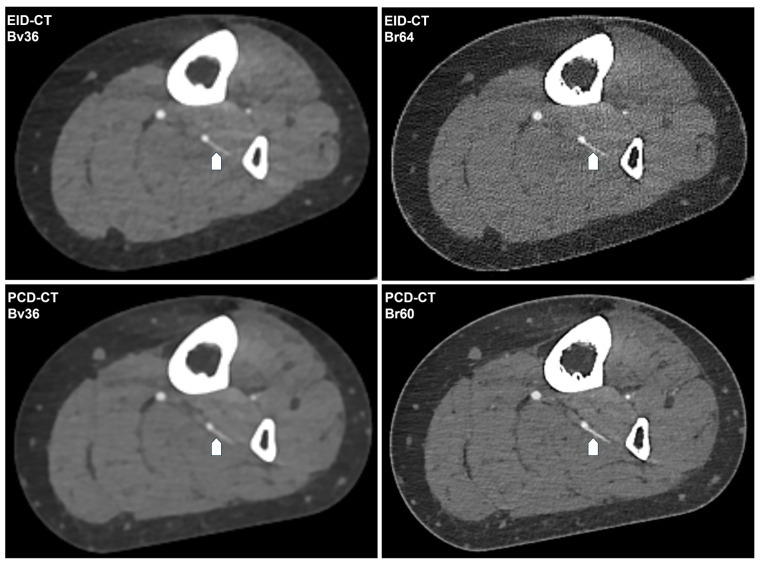
Intraindividual comparison of the CT angiography image quality. The following CT angiography images of a 62-year-old female patient’s lower limb are generated using EID-CT or PCD-CT and reconstructed with the investigated convolution kernels. The same fibular perforator artery is indicated by a white arrow. Window setting: window width 700 HU; window center 100 HU. Bv, body vascular kernel; Br, body regular kernel; EID-CT, energy-integrating CT; HU, Hounsfield units; PCD-CT, photon-counting CT.

Notwithstanding the diminished contrast agent dosage, PCD-CT attains analogous mean luminal attenuation as EID-CT with congruent convolution kernels (e.g., for Bv36, PCD-CT: 452.5 ± 53.6 HU vs. EID-CT: 465.5 ± 99.6 HU; *p* > 0.999). PCD-CT has been demonstrated to exhibit a substantially reduced level of image noise in comparison to EID-CT when employing matched kernels (e.g., for Bv60/64, PCD-CT: 19.4 ± 3.1 HU vs. EID-CT: 33.1 ± 1.7 HU). This results in a significantly higher CNR for PCD-CT for the sharp regular kernel reconstructions (PCD-CT: 22.4 ± 3.5 vs. EID-CT: 14.5 ± 3.8) but not for the soft vascular kernel. Image noise and contrast-to-noise ratio are shown in Figure 2. Table 2 summarizes the objective image parameters of the utilized scanner and kernel. Figure 3 shows the intraindividual comparison of image quality between both scanners and convolution kernels.

### 3.3. Qualitative Image Analysis

**Figure 4:**
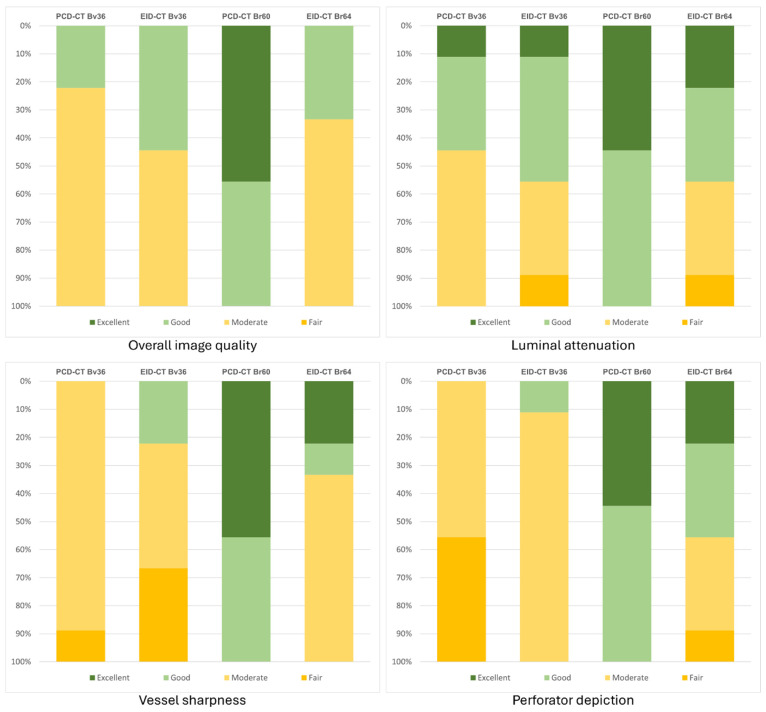
Bar chart graphically representing the subjective image quality ratings. Bv, body vascular kernel; Br, body regular kernel; EID-CT, energy-integrating CT; HU, Hounsfield units; PCD-CT, photon-counting CT.

A subjective assessment reveals minimal and non-significant variations among the two scanner types for reconstructions employing a soft vascular convolution kernel (Bv36). However, when the sharp reconstructions (Br60/64) are taken into consideration, PCD-CT is rated significantly superior in the categories of overall image quality (PCD-CT 5 [4 − 5] vs. EID-CT 3 [3 − 4]; *p* < 0.001), vascular sharpness (PCD-CT 4 [4 − 5] vs. EID-CT 4 [3 − 5]; *p* < 0.001), and delineation of perforating arteries (PCD-CT 5 [4 − 5] vs. EID-CT 3 [3 − 5]; *p* < 0.001). A meticulous evaluation of the subjective assessment of luminal contrast reveals no significant disparities. The interrater agreement was found to range from good to excellent (PCD-CT: 0.58–0.91; EID-CT: 0.75–0.94). Table 3 summarizes the ratings for each scanner type and kernel. Figure 4 displays the distribution of the average subjective ratings.

## 4. Discussion

In this retrospective single-center study, photon-counting CT angiography demonstrated superior visualization of fibular perforator arteries compared with conventional energy-integrating CT angiography, despite a reduced contrast agent volume. While mean luminal attenuation did not differ significantly between techniques, PCD-CTA exhibited markedly lower image noise and a significantly higher contrast-to-noise ratio for sharp kernel reconstructions. These objective enhancements yielded a discernibly superior subjective evaluation of overall image quality, vascular sharpness, and perforator depiction. However, no significant disparities were observed for soft vascular kernels or perceived luminal contrast. The findings, when considered collectively, suggest that the merits of PCD-CT technology become most apparent in scenarios where high-spatial-resolution reconstructions are necessary, thereby underscoring its potential value for preoperative planning in free fibula transplantation. This is particularly salient given the critical importance of reliable depiction of small-caliber perforator arteries in this context.

The findings of the present study demonstrate the potential for enhancing the visualization of perforator arteries through the implementation of PCD-CT, even when employing a conventional protocol. However, further optimization steps would be necessary to fully realize its potential, primarily due to detector-inherent advantages. These include, on the one hand, the use of an ultra-high-resolution mode, which leads to higher spatial resolution and, due to the small pixel effect, to further reduction in image noise [29,30,31], and, on the other hand, the use of even sharper kernels, which are particularly advantageous for small vessels [32,33,34], and the adjustment of the FOV and pixel matrix to transfer the advantage in detector design to image reconstruction [35,36,37,38]. The ultimate objective should be to develop a specific PCD-CT protocol for perforator visualization that could also be adapted to other areas of the body that are relevant for plastic reconstruction.

The intricate process of graft harvesting from the lower leg in free fibula transplantation necessitates meticulous planning to mitigate perioperative risk [39,40,41,42]. The aforementioned approaches have the potential to contribute to a more precise depiction of the perforator arteries, thereby facilitating surgical planning and execution and consequently reducing surgical time due to awareness of possible anatomical variations, predictable incisions, and thus enhanced perioperative safety. This, in turn, enhances graft functionality and ultimately leads to a reduction in morbidity [43,44,45,46].

A small number of studies conducted by other research groups have similarly concentrated on the visualization of perforator arteries using PCD-CT. Lan et al. examined the preoperative visualization of deep inferior epigastric perforator flaps, anterolateral thigh perforator flaps, and superficial circumflex iliac artery perforator flaps. This study investigated 10 patients and revealed that PCD-CTA facilitated the visualization of a greater number of perforator vessels of smaller caliber (approximately +40%) in comparison to conventional CTA. The authors also report a positive effect on surgical time and complication rate and a radiation dose advantage for PCD-CT (approximately −15%), but they do not provide precise details on the CT protocols used [47].

Yalon et al. demonstrated, based on a study population of 52 patients, that PCD-CT facilitates the identification of fibular perforator arteries, with an increase of up to 50% in identified arteries, while concomitantly reducing the required contrast agent dose by half, but in contrast, increasing the radiation dose by approximately 40%. The scan protocols and reconstructions employed in this study distinctly differentiate between PCD-CT and EID-CT. This is evidenced by the utilization of an ultra-high-resolution mode for PCD-CT in lieu of standard resolution, the employment of disparate reconstruction kernels, or the differentiation of the pixel matrix [48]. Consequently, a limited degree of comparison can be made between the two subgroups. Therefore, the objective of our study was to select parameters that were as similar as possible in order to reduce this bias.

The advantage in radiation dose efficiency, as previously demonstrated in earlier studies, cannot be evaluated in the present study due to significantly different scan lengths (PCD-CT: diaphragm to foot; EID-CT: iliac bifurcation to foot) based on clinical indications [18,49]. Representative measurements on the slice level demonstrate that the dosage administered to the lower limbs is more comparable in PCD-CT than in EID-CT, thereby ensuring the comparability of the results obtained in both modalities.

It should be noted that a reduction in contrast agent was not the primary objective of the study. However, protocol changes resulted in an 18% lower dose being applied for PCD-CT. However, given the relative predominance of low-energy photons in comparison to EID-CT, the reduced iodine dose is largely counterbalanced in terms of vascular attenuation [50,51,52,53]. Consequently, PCD-CTA may offer particular benefits in challenging imaging conditions, such as small-caliber vessels or suboptimal contrast enhancement, which are frequently encountered in routine clinical practice.

Consequently, multicenter studies comprising a substantial case number are imperative for the generalizability of the results and determination of the required radiation dose as well as contrast agent quantity. This should be the objective of subsequent research endeavors.

However, it is imperative to acknowledge the limitations of the study. Firstly, the study is of a retrospective, single-center design, utilizing the commercially available PCD-CT at the time of the study. Secondly, given the relatively infrequent nature of the procedure, it was only feasible to establish a relatively modest study cohort. This potential limitation, however, does not detract from the study’s conclusive findings, which hold significant value when comparing the results to those of similar image quality studies. Thirdly, the potential of further optimized PCD-CT protocols remained underutilized. This issue merits further investigation. Fourthly, given the variation in scan lengths, it is not possible to make any definitive statements regarding the potential for dose savings. Finally, no surgical correlation of the perforator vessels was performed; however, preoperative 3D planning and subsequent fibula transplants were based on all included CT angiographies.

## 5. Conclusions

PCD-CTA has been shown to significantly improve the depiction of fibular perforator arteries in comparison with EID-CTA, particularly when high-resolution reconstruction kernels are applied. This enhancement is accomplished despite a reduced contrast agent dosage, attributable to diminished image noise and augmented contrast-to-noise ratios, which collectively yield superior subjective image quality. Consequently, PCD-CTA demonstrates considerable promise in enhancing preoperative planning for free fibula transplantation.

## Figures and Tables

**Table 1:** Scan parameters and image reconstruction.

Parameter	PCD-CT	EID-CT
Collimation	140 × 0.4 mm	192 × 0.6 mm *
Tube voltage	140 kVp	70/80/90 kVp ^#^
Tube current	50 mAs	285/171/120 mAs
Image quality index	156	-/-
Rotation Time	0.5 s	0.5 s
Pitch	0.40	0.45
Convolution kernels	Bv36/Br60	Bv36/Br64
Slice thickness/increment	1.0 mm	1.0 mm
Iterative reconstruction	QIR Level 3	ADMIRE Level 3

ADMIRE, ADvanced Modeled Iterative REconstruction; EID-CT, energy-integrating detector computed tomography; PCD-CT, photon-counting detector computed tomography; QIR, Quantum Iterative Reconstruction. * z-flying focal spot 2 × 96 mm × 0.6 mm. ^#^ ATVS, Automatic Tube Voltage Selection.

**Table 2:** Overview of objective image parameters, radiation and contrast agent dose.

Parameter	Kernel	PCD-CT	EID-CT	*p*-Values *
Arterial attenuation [HU]	Bv36	452.5 ± 53.6	465.5 ± 99.6	0.375
	Br60/64	524.5 ± 69.9	527.4 ± 119.3	0.529
Image Noise [HU]	Bv36	5.5 ± 0.5	6.8 ± 1.0	**<0.001**
	Br60/64	21.1 ± 1.0	32.9 ± 1.6	**<0.001**
Contrast-to-noise ratio	Bv36	73.3 ± 10.0	62.7 ± 17.9	0.105
	Br60/64	22.4 ± 3.5	14.5 ± 3.8	**<0.001**
CTDI_Vol_ [mGy]	-/-	7.07 ± 0.90	6.40 ± 2.97	0.491
Contrast agent dose ^#^ [mL]	-/-	90 ± 0	110 ± 0	**<0.001**

Bv, body vascular kernel; Br, body regular kernel; CTDI_Vol_, computed tomography dose index; EID-CT, energy-integrating CT; HU, Hounsfield units; PCD-CT, photon-counting CT. * Comparison of PCD-CT versus EID-CT of the same row, significant *p*-values are highlighted in bold font. ^#^ Imeron^®^ 350 mg iodine/mL.

**Table 3:** Overview of the subjective image quality ratings.

Parameter	Kernel	PCD-CT	EID-CT	*p*-Values *
Overall image quality	Bv36	3 [3 − 4]	3 [3 − 4]	>0.999
	Br60/64	5 [4 − 5]	3 [3 − 4]	**<0.001**
Luminal attenuation	Bv36	3 [3 − 4]	4 [3 − 4]	>0.999
	Br60/64	4 [4 − 5]	4 [3 − 5]	0.290
Vessel sharpness	Bv36	3 [2 − 3]	3 [3 − 3]	0.463
	Br60/64	4 [4 − 5]	4 [3 − 5]	**0.031**
Perforator artery depiction	Bv36	3 [3 − 3]	3 [2 − 4]	>0.999
	Br60/64	5 [4 − 5]	3 [3 − 5]	**0.019**

Bv, body vascular kernel; Br, body regular kernel; EID-CT, energy-integrating CT; PCD-CT, photon-counting CT. * Comparison of PCD-CT versus EID-CT of the same row; significant *p*-values are highlighted in bold font.

## Data Availability

The datasets generated and/or analyzed during this study are not publicly available, as CT data and DICOM headers contain patient information. Anonymized data can be obtained upon reasonable request from the corresponding author.

## Abbreviations

PCD-CT	photon-counting detector CT
PCD-CTA	photon-counting detector CT angiography
EID-CT	energy-integrating detector CT
EID-CTA	energy-integrating detector CT angiography
FT	free fibula transplantation
FPA	perforator arteries of the fibular artery
IQL	image quality level
QIR	quantum iterative reconstruction
ADMIRE	advanced modeled iterative reconstruction
ROI	region of interest
IQR	interquartile ratio
CNR	contrast-to-noise ratio
